# Quantification of morpho-hemodynamic changes in unruptured intracranial aneurysms with irregular pulsation during the cardiac cycle using 4D-CTA

**DOI:** 10.3389/fneur.2024.1436086

**Published:** 2024-07-23

**Authors:** Shiyao Chen, Wen Zhang, Yunzhang Cheng, Guohui Wang, Nan Lv

**Affiliations:** ^1^School of Health Science and Engineering, University of Shanghai for Science and Technology, Shanghai, China; ^2^Shanghai Interventional Medical Device Engineering Technology Research Center, University of Shanghai for Science and Technology, Shanghai, China; ^3^Shanghai HeartCare Medical Technology Corporation Limited, Shanghai, China; ^4^Cerebrovascular Disease Center, First Affiliated Hospital of Naval Military Medical University, Changhai Hospital of Shanghai, Shanghai, China

**Keywords:** intracranial aneurysms, irregular pulsation, 4D-CTA, computed fluid dynamics, hemodynamics, rupture risk

## Abstract

**Background and purpose:**

Previous studies predicting the rupture risk of intracranial aneurysms (IAs) have predominantly utilized static imaging data, overlooking the dynamic blood flow and biomechanical properties of the aneurysm wall. Irregular pulsation detected by 4D-CTA is a potential predictor of aneurysm rupture, albeit with uncertain clinical significance. This study aimed to analyze the changes in morpho-hemodynamic characteristics of IAs during the cardiac cycle to elucidate the dynamic changes and the associated hemodynamic mechanisms.

**Methods:**

A retrospective review was conducted on the 4D-CTA data of IA patients between January 2017 and September 2019. R-R intervals were segmented into 20-time phases, reconstructing 20 CT datasets to identify irregular pulsation and extract 3D aneurysm models. Computational fluid dynamics (CFD) simulations analyzed hemodynamic parameters such as oscillatory shear index (OSI) and relative residence time (RRT). Changes in morpho-hemodynamic characteristics were quantified in terms of the absolute change (parameter*) and relative change rate (parameter%). Rupture risk was assessed using the rupture resemblance model (RRS).

**Results:**

Eleven UIAs from 10 patients were finally included, with five aneurysms showing irregular pulsation (45.45%). No significant differences in morpho-hemodynamic characteristics were observed between aneurysms with or without irregular pulsation. More remarkable changes in aneurysm size (size*: 0.59 ± 0.14 mm vs. 0.32 ± 0.12 mm, *p* = 0.010; size%: 10.49% ± 1.43% vs. 3.95% ± 1.79%, *p* < 0.001), volume (volume%: 13.72% vs. 6.39%, *p* = 0.009), OSI (OSI*: 0.02 ± 0.01 vs. 0.004 ± 0.005, *p* = 0.004; OSI%: 200% vs. 12.50%, *p* = 0.004) and RRT (RRT%: 97.14% vs. 43.95, *p* = 0.052) over the cardiac cycle were significantly linked to irregular pulsation. Aneurysms with irregular pulsation demonstrated a more unfavorable hemodynamic environment during the cardiac cycle, irrespective of the predicted rupture risk. Furthermore, irregular pulsation at the aneurysm dome exhibited higher hemodynamic instability than at the sidewall.

**Conclusion:**

Irregular pulsation may indicate hemodynamic instability within the aneurysm, leading to an increased rupture risk in the area where irregular pulsation occurs. This proof-of-concept study could enhance understanding of dynamic changes in UIAs during the cardiac cycle and the underlying hemodynamic mechanisms.

## Introduction

Accurately assessing the risk of rupture in unruptured intracranial aneurysms (UIAs) is essential for personalized management (1). IA is a cerebrovascular disease that results from changes to the arterial wall due to abnormal blood flow conditions (2), with hemodynamic stress playing an indispensable role in its formation, progression, and even rupture (3). Computational fluid dynamics (CFD) is a widely accepted method for simulating hemodynamics *in vitro* (4). While previous studies have used static imaging data like conventional computed tomography angiography (CTA) to analyze hemodynamics and predict aneurysm rupture risk (5, 6), the lack of consideration for dynamic blood flow and the biomechanical properties of the aneurysmal wall has limited the clinical translation of this knowledge (7).

Four-dimensional CTA (4D-CTA) is an advanced imaging technique that combines the non-invasiveness of traditional CTA with the dynamic acquisition of digital subtraction angiography (DSA) to provide detailed and comprehensive information about vascular structure and blood flow dynamics (8). Studies have demonstrated the ability of 4D-CTA to capture the cardiac cycle-related motion of the aneurysm wall reliably and suggested irregular pulsation could be a new risk indicator for aneurysm rupture (9–12). Inspired by the study of Ishida et al. (13), who proposed that the pulsatile motion of the aneurysmal wall detected by 4D-CTA may not only indicate changes in the shape of the aneurysm but also provide dynamic information about the hemodynamics within the aneurysm. Investigations on the morpho-hemodynamic dynamics of IAs may provide new insights into the hemodynamic mechanisms of aneurysm rupture. To the best of our knowledge, few studies have considered the effect of arterial and aneurysmal deformation, pulsatile pressure, and flow velocity on the results of CFD simulations. Hence, we investigated the morpho-hemodynamic characteristics and their changes in aneurysms during the cardiac cycle using 4D-CTA and CFD techniques to understand better the relationship between the dynamic changes of IAs and hemodynamics.

## Materials and methods

### Patients

This retrospective study received approval from the Institutional Review Board of Changhai Hospital. The requirement for written informed consent was waived, and patient information was anonymized and de-identified before analysis.

**Figure 1:**
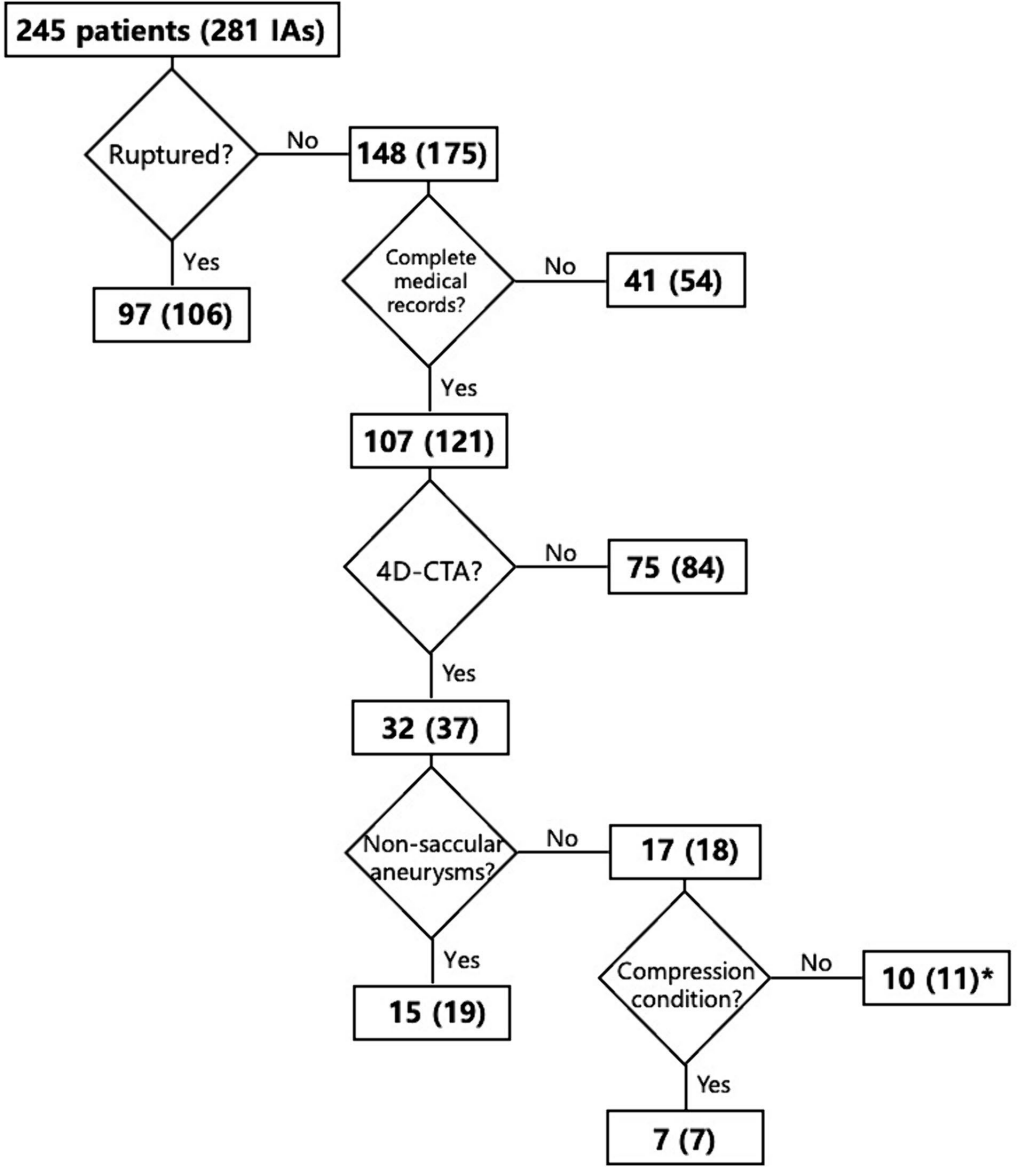
Flowchart of patient selection based on inclusion and exclusion criteria.

Patients with IAs from January 2017 to September 2019 were reviewed. The inclusion criteria were: (1) UIAs, (2) patients with complete medical records, and (3) patients with 4D-CTA data. The exclusion criteria were: (1) patients reluctant to participate in this study, (2) ruptured or non-saccular (fusiform, dissecting, and infectious) IAs, and (3) patients presenting with compression symptoms due to the proximity of the aneurysm to surrounding tissues or large aneurysms. Based on the inclusion and exclusion criteria, the flowchart for the selection of patients is shown in Figure 1.

### 4D-CTA data acquisition

The imaging protocol for electrocardiogram (ECG)-gated CTA scanning involved the use of a 320-row CT scanner (Aquilion ONE, Toshiba, Japan) in volumetric imaging mode. Initially, a test bolus with a 10-mL non-ionic contrast medium was injected at a rate of 5 mL/s to determine the delay time. Subsequently, a 50 mL iodinated contrast medium was intravenously administrated at a rate of 5 mL/s, along with 20 mL saline. 4D-CTA scanning commenced after an appropriate delay time determined by the injection test. The scan parameters include a 120 kV tube voltage, 260 mA tube current, a gantry rotation speed of 0.275 s per turn, and a *z*-coverage of 160 mm. The scan time was at least one heartbeat, and the in-plane resolution was 0.31 mm.

The source data from the 4D-CTA were transferred to an image processing workstation (Vitrea 2 software 6.02, Toshiba, Japan) for ECG-gated reconstruction of the cardiac cycle. The R-R intervals were segmented into 20-time phases at 5% intervals to create 20 CT data packages with an image matrix size of 512 × 512 and a slice thickness of 0.5 mm. The reconstructed datasets were stored in DICOM format for subsequent processing.

### Criteria for determining irregular pulsation in aneurysms

Irregular pulsation on the aneurysmal wall is a focal protuberance observed in at least three consecutive frames within the 20 CT images corresponding to the 20-time phases in the R-R interval obtained by 4D-CTA (9, 10). Another form of aneurysm wall motion during the cardiac cycle, termed global dilation or overall dilation, was identified. A neuroradiologist and a neurosurgeon consistently confirmed the type of aneurysm wall motion through a comprehensive review of the 4D-CTA images.

### Reconstruction of 3D models of patient-specific aneurysms

**Figure 2:**
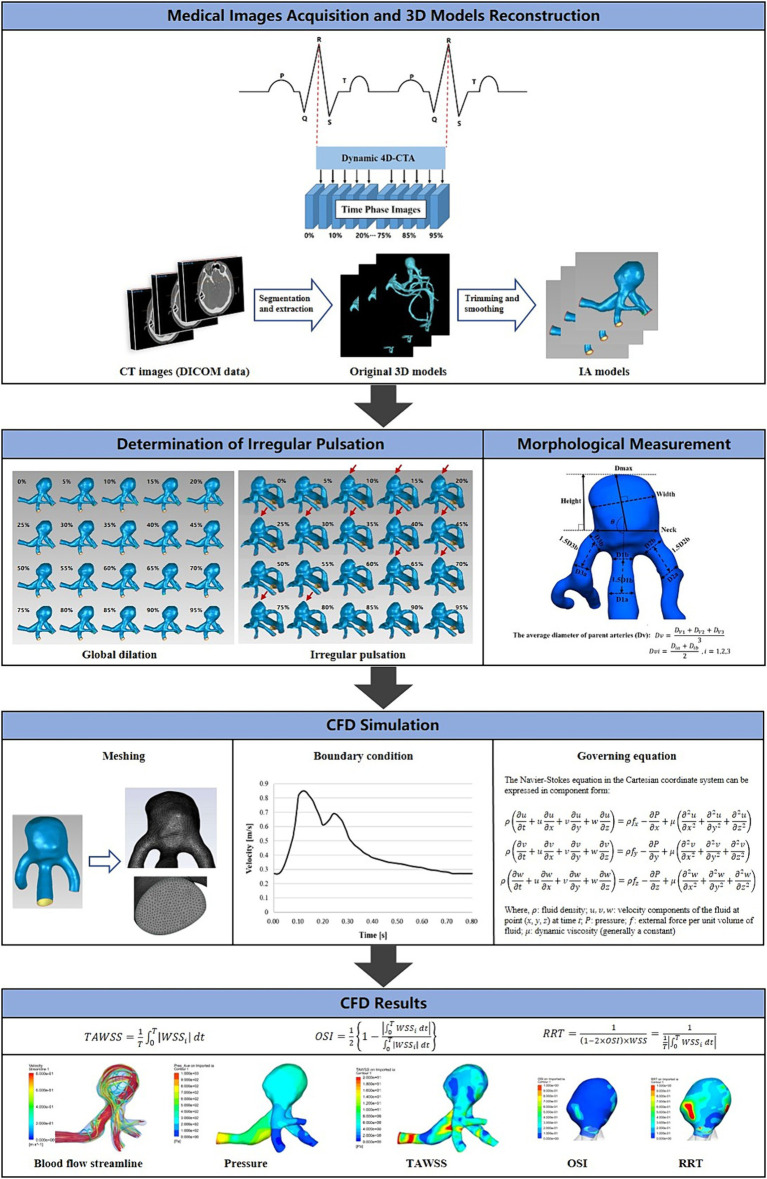
The main workflow diagram of this study. The fundamental operations of this study were divided into five sections: the acquisition of 4D-CTA images of intracranial aneurysms, the determination of the type of aneurysm wall motion during the cardiac cycle, the reconstruction of 3D aneurysm models, the measurement of morphological features, and the CFD hemodynamic analysis.

Figure 2 depicts the main workflow of this study is depicted. Each DICOM file was subjected to analysis using Mimics software (Materialise Inc.) to extract aneurysm models corresponding to each specific time phase of the cardiac cycle. A threshold of cerebrovascular tissue was established to differentiate the aneurysm from surrounding structures like the skull. In addition, redundant small branching arteries were eliminated, and ultimately, the aneurysm model was generated through 3D calculation (14, 15). The resulting 3D structure was exported in stereolithography (STL) format for further analysis.

### Element meshing

The ICEM CFD 2020R2 (ANSYS Inc., Canonsburg, PA, United States) was used to generate volume grids for conducting fluid dynamics simulations. The aneurysm models were discretized using an unstructured tetrahedral mesh type. Boundary layer meshing was progressively encrypted in the fluid domains adjacent to the vessel wall. The initial boundary layer had a thickness of 0.02 mm with a growth ratio of 1.2, resulting in 5 layers. The final number of meshes generated was in the range of 800,000 to 1,200,000.

### Boundary conditions

CFD analysis was performed using FLUENT 2020R2 (ANSYS Inc., Canonsburg, PA, United States). The calculations were based on the Navier–Stokes formulations. Blood was assumed to be an incompressible and Newtonian fluid, with a density of 1,056 $\mathrm{kg}/m^{3}$ and a dynamic viscosity of 0.0035 Pa⋅s . Rigid wall boundaries with no-slip conditions were considered (16). Due to the low Reynolds number features of arterial blood flow, it was treated as laminar flow (17). The pulsatile flow at the inlet was derived from measurements obtained using transcranial Doppler ultrasound (TCD) from a healthy adult volunteer’s internal carotid arteries (ICA). The flow data was processed and fitted using MATLAB 2016b software. The outlets were defined with a zero static pressure condition. The entire cycle of 0.8 s was discretized using a time step size of 0.001 s. Three continuous cardiac cycles were simulated, and the output was taken from the last cycle.

### Morphological and hemodynamic parameters

Rupture-related morphological and hemodynamic features were analyzed, including aneurysm size, volume, and SR (18), as well as blood flow velocity, flow pattern, pressure, wall shear stress (WSS), oscillatory shear index (OSI), and relative residence time (RRT) (19). Time-averaging was employed for each hemodynamic parameter to assess the hemodynamic variations in each aneurysm as a consequence of the morphological changes during the cardiac cycle.

### Quantification of rupture-related parameters

We applied the quantification method to other morphological-hemodynamic features based on the definitions of the expansion volume $\left(V_{\text{max}}-V_{\text{min}}\right)$ and expansion rate $\left(\frac{V_{\text{max}}-V_{\text{min}}}{V_{\text{min}}}\right)$ during the cardiac cycle proposed by Kuroda et al. (20). Thus, changes in parameters over the cardiac cycle were quantified by (1) absolute change (*), which is the difference between the maximum and minimum values of the parameter over the cardiac cycle, and (2) relative rate of change (%), which is the ratio of the difference between the maximum and minimum values to the minimum value of the parameter over the cardiac cycle.

### Rupture risk assessment

The rupture resemblance model (RRS) is a scoring system used to estimate the risk of aneurysm rupture, including morphological (RRS_M_), hemodynamic (RRS_H_), and combined morphological-hemodynamic (RRS_C_) models (21). The RRS is calculated by dividing the odds by one plus the odds, and the odds are calculated below:


$${\mathrm{Odd}}_{M}=e^{1.09\cdot SR-2.99}\#\left(1\right)$$

$${\mathrm{Odd}}_{H}=e^{-0.73\cdot WSS+2.86\cdot OSI-0.12}\#\left(2\right)$$

$${\mathrm{Odd}}_{C}=e^{0.73\cdot SR-0.45\cdot WSS+2.19\cdot OSI-2.09}\#\left(3\right)$$

In previous studies, each parameter used in the RRS was scaled from 0 to 10 based on the distribution of the previous population. The RRS threshold is considered to be 30%, with higher values indicating an increased risk of rupture (6).

### Agreement test

Operator 1 and Operator 2 independently completed the 3D reconstruction of 5 aneurysms randomly selected from the 11 UIAs three months later. The intra- and inter-observers’ agreements were analyzed by calculating intra-class correlation coefficients (ICCs), to assess the reproducibility of 3D reconstruction in quantifying dynamic changes of aneurysms, which were good (ICC > 0.8).

### Statistical analysis

Statistical analysis was conducted using IBM SPSS Statistics 26 software. Measured variables were expressed in numbers. The normality of continuous parameters was determined using the Shapiro–Wilk test. The values were presented as mean ± standard deviation (SD) or median values with the range, depending on whether they followed a normal distribution. The independent-sample *t*-test or the Mann–Whitney U test was used for correlation analysis. Set α= 0.05 as the significance level and *p* < 0.05 (two-sided) as statistically different.

## Results

A total of 10 patients with 11 UIAs were finally included in this study. Table 1 concludes the basic characteristics of patients and aneurysms. Of the ten patients, there were six females and four males, and the ages ranged from 38 to 76 years, with a mean age of 62.20. Of the 11 UIAs, 5 showed cardiac cycle-related irregular pulsation (45.45%). Five aneurysms were located at the middle cerebral artery (45.45%), three at the vertebral artery (27.27%), one at the internal carotid artery (9.09%), one at the posterior communicating artery (9.09%), and one at the anterior communicating artery (9.09%). The basic characteristics were not correlated with irregular pulsation.

**Table 1:** Basic characteristics of the patients and aneurysms.

Patients (No.)	Aneurysm (No.)	Age (Year)	Gender	Location	Dmax (mm)	Volume (mm^3^)	SR	RRS_M_	RRS_H_	RRS_C_	Irregular pulsation
1	1	52	M	MCA	9.30 ± 0.08	339.70	3.76	75.52%	34.28%	58.74%	N
2	2	71	F	MCA	6.55 ± 0.18	86.08	2.88	53.51%	42.67%	47.68%	Y
3	3	60	F	AcomA	3.59 ± 0.07	44.56	1.41	18.90%	38.95%	22.25%	N
4	4	72	M	MCA	6.32 ± 0.08	178.42	2.21	36.51%	31.69%	29.88%	N
5	5	76	F	MCA	4.56 ± 0.14	68.55	1.34	17.64%	32.90%	18.67%	Y
6	6	69	F	ICA	16.50 ± 0.14	961.16	3.40	67.11%	41.79%	56.57%	N
	7			PcomA	7.72 ± 0.06	68.14	1.61	22.52%	36.84%	23.71%	N
7	8	71	F	MCA	13.85 ± 0.10	1022.90	4.84	90.71%	44.78%	80.03%	N
8	9	49	F	VA	3.94 ± 0.14	33.96	1.12	14.75%	43.11%	20.59%	Y
9	10	64	M	VA	8.48 ± 0.25	74.96	1.73	24.70%	43.91%	28.79%	Y
10	11	38	M	VA	6.41 ± 0.17	86.17	1.55	21.55%	45.25%	27.08%	Y

M, male; F, female; MCA, middle cerebral artery; AcomA, anterior communicating artery; ICA, internal carotid artery; PcomA, posterior communicating artery; VA, vertebral artery; Dmax, aneurysm maximum diameter; SR, size ratio; RRS_M_, rupture resemblance model using morphological parameters; RRS_H_, rupture resemblance model using hemodynamic parameters; RRS_C_, rupture resemblance model using morphological-hemodynamic parameters; Y, yes (indicating aneurysms with irregular pulsation); N, no (indicating aneurysms with global dilation).

### Differences in morpho-hemodynamic characteristics between aneurysms with irregular pulsation and global dilation

As shown in Table 2, there were no differences in aneurysm size, volume, pressure, WSS, OSI, and RRT between aneurysms with irregular pulsation and those with overall dilatation. Moreover, the RRS models were also not significantly associated with irregular pulsation.

**Table 2:** Differences in hemodynamic characteristics and their changes over the cardiac cycle between aneurysms with irregular pulsation and global dilation.

Group parameters	Irregular pulsation (*N* = 5)	Global dilation (*N* = 6)	*p*
Size (mm)	5.99 ± 1.80	9.55 ± 4.82	0.141
Size* (mm)	0.59 ± 0.14	0.32 ± 0.12	**0.010**
Size%	10.49% ± 1.43%	3.95% ± 1.79%	**<0.001**
Volume (mm^3^)	74.96 (33.96, 86.17)	259.06 (44.56, 1022.90)	0.247
Volume* (mm^3^)	9.55 (4.34, 23.72)	15.98 (4.21, 53.77)	0.537
Volume%	13.72% (11.44, 30.67%)	6.39% (3.98, 11.49)	**0.009**
Pressure (Pa)	464.55 ± 120.20	479.45 ± 259.24	0.909
Pressure* (Pa)	353.56 ± 169.63	211.62 ± 128.74	0.148
Pressure%	114.87% ± 57.09%	60.65% ± 28.57%	0.105
TAWSS (Pa)	0.39 ± 0.32	0.59 ± 0.30	0.327
TAWSS* (Pa)	0.32 ± 0.16	0.38 ± 0.19	0.605
TAWSS%	147.25% ± 39.54%	103.26% ± 32.54%	0.083
OSI	0.02 (0.01, 0.03)	0.01 (0.01, 0.04)	0.429
OSI*	0.02 ± 0.01	0.004 ± 0.005	**0.004**
OSI%	200% (100, 275%)	12.50% (0, 33.33%)	**0.004**
RRT	0.46 (0.16, 1.81)	0.26 (0.12, 0.78)	0.247
RRT*	0.34 (0.07, 3.01)	0.09 (0.02, 0.43)	0.247
RRT%	97.14% (52.63, 376.25%)	43.95 (18.18, 70.49%)	0.052^b^
RRS_M_	21.55% (14.75, 53.51%)	51.81% (18.90, 90.71%)	0.126
RRS_H_	41.57% ± 4.94%	38.04% ± 4.44%	0.243
RRS_C_	28.56% ± 11.50%	45.32% ± 23.58%	0.166

Continuous values conforming to a normal distribution are expressed as mean ± SD, whereas those that deviate from a normal distribution are represented by the median (range). TAWSS, time-averaged wall shear stress; OSI, oscillatory shear index; RRT, relative residence time; (parameter)*, the absolute change of the parameter during the cardiac cycle; (parameter)%, the relative change rate of the parameter; RRS_M_, rupture resemblance model using morphological parameters; RRS_H_, rupture resemblance model using hemodynamic parameters; RRS_C_, rupture resemblance model using morphological-hemodynamic parameters. The bold values indicate statistical significance, and superscript ‘b’ indicates a statistically significant trend.

### Differences in cardiac cycle-related morpho-hemodynamic changes between aneurysms with irregular pulsation and global dilation

As Table 2 illustrates, irregular pulsation was significantly correlated with more remarkable changes in aneurysm size (size*: 0.59 ± 0.14 mm vs. 0.32 ± 0.12 mm, *p* = 0.010; size%: 10.49 ± 1.43% vs. 3.95% ± 1.79%, *p* < 0.001), volume (volume%: 13.72% vs. 6.39%, *p* = 0.009), OSI (OSI*: 0.02 ± 0.01 vs. 0.004 ± 0.005, *p* = 0.004; OSI%: 200% vs. 12.50%, *p* = 0.004), and RRT (RRT%: 97.14% vs. 43.95%, *p* = 0.052) during the cardiac cycle.

### Subgroup analysis

In this part, the hemodynamics and their variations during the cardiac cycle of four UIAs were compared, based on the presence of irregular pulsation and rupture risk stratification, to examine the hemodynamics of different types of aneurysms and their changes throughout the cardiac cycle. They were: (1) high rupture risk without irregular pulsation, (2) high rupture risk with irregular pulsation, (3) low rupture risk without irregular pulsation, and (4) low rupture risk with irregular pulsation.

**Figure 3:**
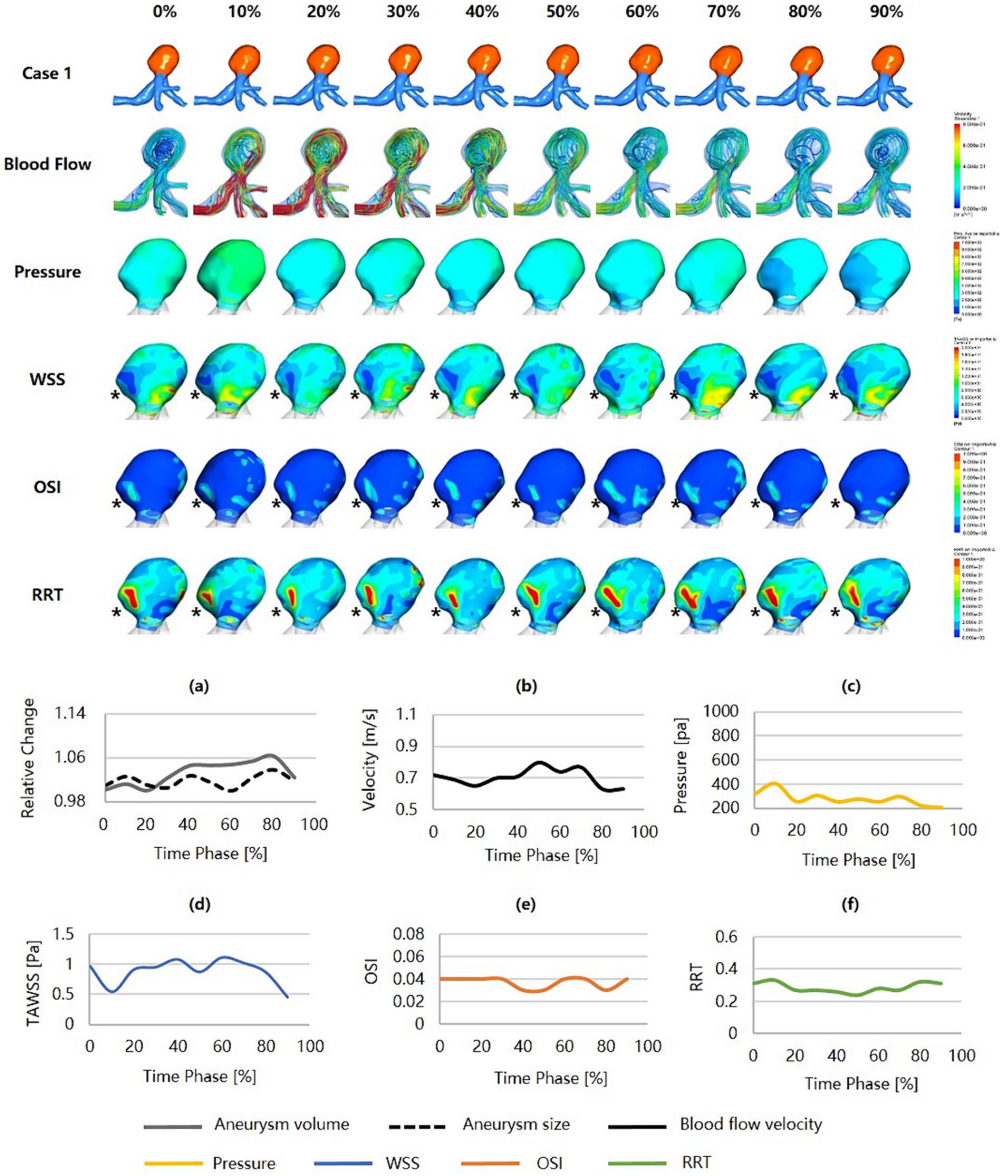
Hemodynamics and their variations during the cardiac cycle of an MCA aneurysm with a high rupture risk but no irregular pulsation. **(a–f)** are the aneurysm size, volume, blood flow velocity, pressure, WSS, OSI, and RRT, respectively, for the aneurysm during the cardiac cycle (black snowflakes indicate the area of aneurysm wall with unfavorable hemodynamic conditions). MCA, middle cerebral artery; WSS, wall shear stress; OSI, oscillatory shear index; RRT, relative residence time.

Figure 3 was an unruptured MCA aneurysm without cardiac cycle-related irregular pulsation, assessed as a high risk of rupture (RRS_M_ = 75.52%, RRS_H_ = 34.28%, RRS_C_ = 58.74%). Morphologically, this was a large aneurysm (size = 9.30 mm) with a high SR (SR = 3.76) but without shape irregularity. Regarding hemodynamics, neither blood flow nor pressure was significantly abnormal, and they varied smoothly throughout the cardiac cycle. However, the region with a more protruding aneurysmal wall exhibited markedly lower WSS, higher OSI, and higher RRT (shown as black snowflakes). In particular, this region consistently presented throughout the cardiac cycle.

**Figure 4:**
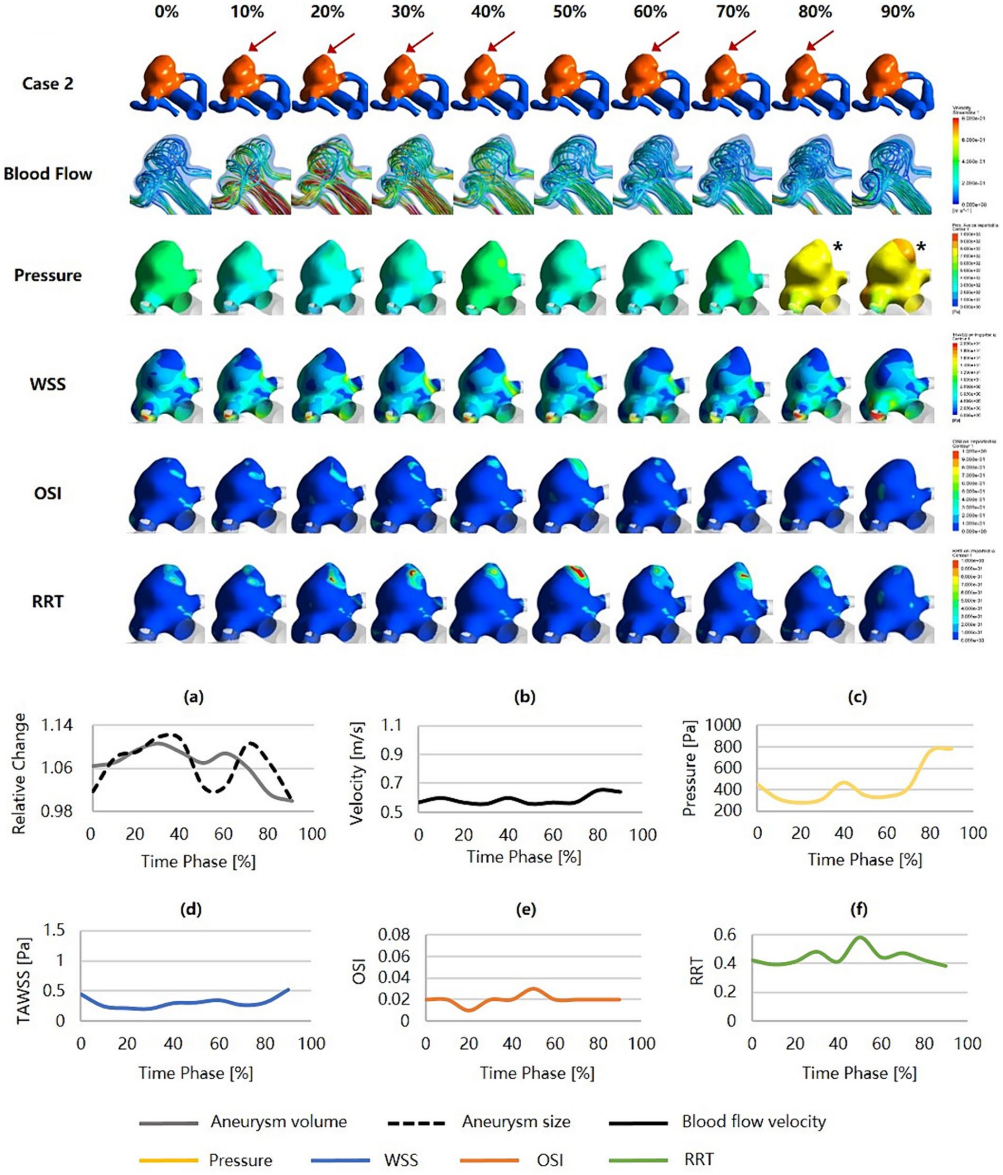
Hemodynamics and their variations during the cardiac cycle of an MCA aneurysm with a high rupture risk and irregular pulsation. **(a–f)** are the aneurysm size, volume, blood flow velocity, pressure, WSS, OSI, and RRT, respectively, for the aneurysm during the cardiac cycle (red arrows indicate the location of irregular pulsation, and black snowflakes denote the region with significantly high pressure). MCA, middle cerebral artery; WSS, wall shear stress; OSI, oscillatory shear index; RRT, relative residence time.

Figure 4 was an unruptured MCA aneurysm exhibiting irregular pulsation during the cardiac cycle. RRS suggested a high rupture risk (RRS_M_ = 53.51%, RRS_H_ = 42.67%, RRS_C_ = 47.68%). The pulsation occurred at the aneurysm dome (indicated by the red arrow). Although it was a small aneurysm (size = 6.55 mm), it featured an irregular shape and a high SR (SR = 2.88). Significant changes in aneurysm size and geometry were observed during the cardiac cycle. The blood flow within the aneurysm sac was complex and unstable. Slow blood flow and vortex flow were presented. The wall of the aneurysm dome (i.e., the area of irregular pulsation) was characterized by significantly higher pressure, lower WSS, higher OSI, and higher RRT than the rest of the aneurysm wall. Particularly towards the end of the cardiac cycle, the pressure in the aneurysm reached its peak, and so did in the aneurysm dome (as indicated by the black snowflakes).

**Figure 5:**
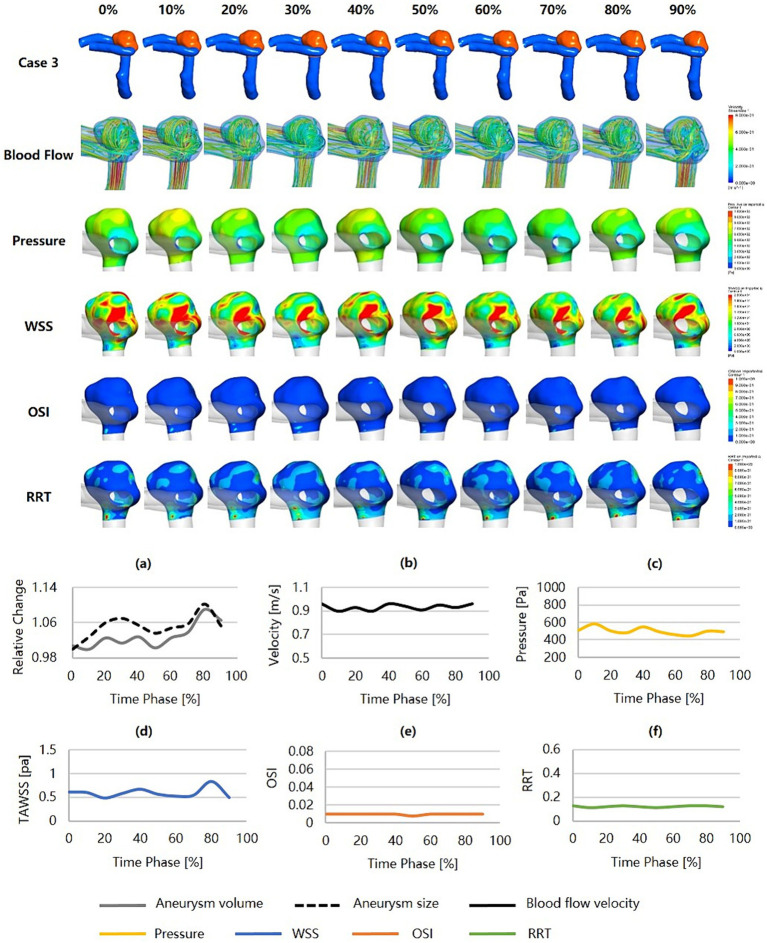
Hemodynamics and their variations during the cardiac cycle of an AcomA aneurysm with a low rupture risk and no irregular pulsation. **(a–f)** are the aneurysm size, volume, blood flow velocity, pressure, WSS, OSI, and RRT, respectively, for the aneurysm during the cardiac cycle. AcomA, anterior communicating artery. WSS, wall shear stress; OSI, oscillatory shear index; RRT, relative residence time.

Figure 5 was an unruptured AcomA aneurysm (Dmax = 3.59 mm, SR = 1.41) that was considered a relatively low risk of rupture (RRS_M_ = 18.90%, RRS_H_ = 33.27%, RRS_C_ = 19.73%), and no irregular pulsation presented over the cardiac cycle. From a morphological perspective, the aneurysm carried a low risk of rupture and a low degree of dynamic variation over the cardiac cycle. From a hemodynamic perspective, the blood flow velocity was high, and there were relatively intense and concentrated inflow jets with high pressure at the inflow impingement. The aneurysm wall covered high WSS, low OSI, and low RRT throughout the cardiac cycle, with minimal variation.

**Figure 6:**
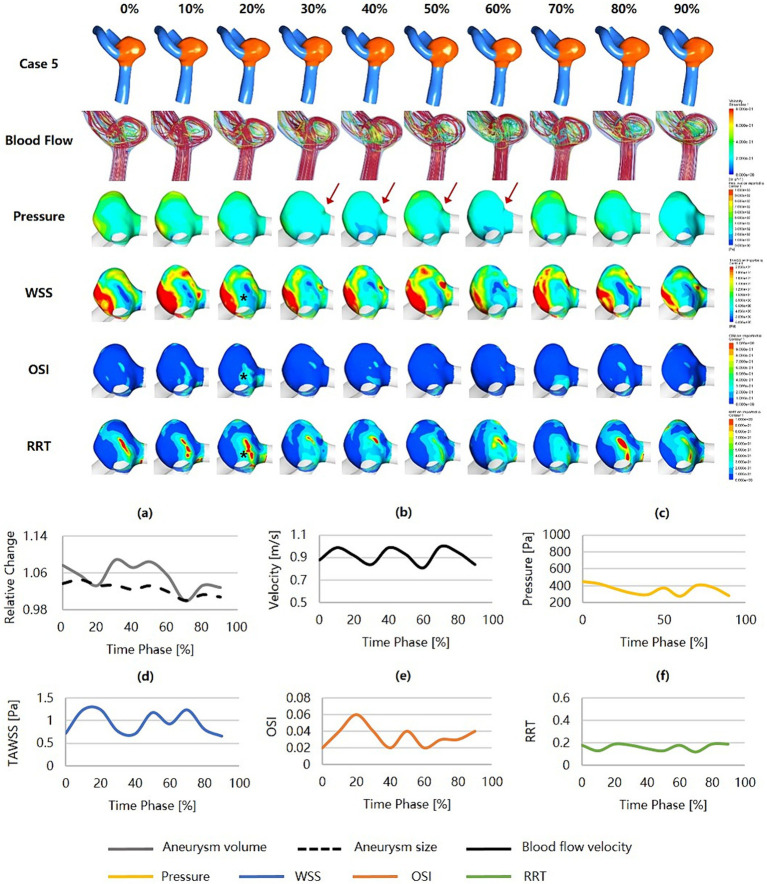
Hemodynamics and their variations during the cardiac cycle of an MCA aneurysm with a low rupture risk but irregular pulsation. **(a–f)** are the aneurysm size, volume, blood flow velocity, pressure, WSS, OSI, and RRT, respectively, for the aneurysm during the cardiac cycle (red arrows indicate the location of irregular pulsation). MCA, middle cerebral artery; WSS, wall shear stress; OSI, oscillatory shear index; RRT, relative residence time.

Figure 6 was an unruptured MCA aneurysm (Dmax = 4.56 mm, SR = 1.34) with irregular pulsation (as the red arrows show), which was estimated to have a relatively low risk of rupture (RRS_M_ = 17.64%, RRS_H_ = 32.90%, RRS_C_ = 18.67%). The blood flow velocity was high, but the flow pattern was not complex. The inflow jets were strong and concentrated, and the inflow impingements to the aneurysm wall were characterized by higher pressure and substantially higher WSS. This aneurysm held low and smoothly varying pressure and RRT, while WSS and OSI were substantially higher and more variable. The hemodynamic environment was not significantly worse at the irregular pulsation, as indicated by only a higher WSS.

## Discussion

This study used 4D-CTA and CFD techniques to simulate the hemodynamics and quantified hemodynamic changes during the cardiac cycle in 11 UIAs to investigate the hemodynamic mechanism of aneurysm dynamic changes. Ishida et al. argued that the pulsatile motion of the aneurysm wall depicted by 4D-CTA is the result of visualizing the interaction between the aneurysm wall and hemodynamics (13), taking into account not only the biomechanical properties of the aneurysm wall but also the effects of pulsatile blood flow. Given that the hemodynamics depend on aneurysm morphology under the rigid-wall assumption, and TCD data from a healthy volunteer was employed as the inlet condition in the CFD boundary conditions, time averaging was conducted for each hemodynamic parameter to analyze the hemodynamic differences in each aneurysm resulting from morphological changes throughout the cardiac cycle. As far as we know, this is the first study to explain the dynamics of UIAs during the cardiac cycle by 4D-CTA and CFD simulations and to explore the morpho-hemodynamic mechanisms of aneurysm dynamics.

It was found that aneurysms with irregular pulsation exhibited significantly greater changes in aneurysm size, volume, OSI, and RRT over the cardiac cycle. The findings suggest that the morphological and hemodynamic characteristics of aneurysms indeed change during the cardiac cycle, and the degrees of changes vary considerably between aneurysms with and without irregular pulsation.

In addition, the hemodynamics and their variations over the cardiac cycle of four aneurysm categories based on the types of aneurysm wall motion and the rupture risk estimated by RRS were further analyzed. We found no correlation between irregular pulsation and RRS. Even though aneurysms were assessed with a higher rupture risk, those without irregular pulsations exhibited a more stable hemodynamic condition than those with irregular pulsations during the cardiac cycle. In cases of UIAs with irregular pulsation, two small and bifurcated MCA aneurysms were analyzed. In the aneurysm with a higher rupture risk, irregular pulsation occurred at the top of the aneurysm wall. The region with irregular pulsation featured an adverse hemodynamic environment with higher pressure, lower WSS, higher OSI, and higher RRT. Several CFD studies have demonstrated a correlation between low WSS (or larger area of low WSS) at the aneurysm dome and the subsequent aneurysm rupture (22, 23). Low WSS and high OSI may trigger inflammatory cell-mediated destructive remodeling of the aneurysm wall (24). Kadasi et al. (25) combined surgical findings and discovered that thin walls were linked to low WSS. Therefore, irregular pulsation at the aneurysm dome may disrupt normal blood flow patterns, resulting in areas of lower WSS, which can lead to the development of inflammation, endothelial dysfunction, and weakening of the aneurysm wall, which may increase the risk of aneurysm rupture. In contrast, irregular pulsation in aneurysm with a lower rupture risk occurred at the sidewall. The hemodynamic condition was not worse, but only the WSS was increased. Meng et al. (24) hypothesized that high WSS could cause cellular matrix degeneration and apoptosis degeneration, so even tiny aneurysms may rupture. Notably, the aneurysm was directly impacted by the strong and concentrated inflow against the aneurysmal wall. Cebral et al. (26, 27) reported that a more significant concentrated inflow and small impingement regions were associated with ruptured IAs. Thus, the rupture risk of this type of aneurysm should not be ignored. Nevertheless, the present study failed to investigate whether the aneurysm dome, susceptible to direct impingement of blood inflow or irregular pulsation in the sidewall, may have a higher rupture risk because of the limited datasets.

The shape of aneurysms changes during the cardiac cycle due to the interaction between arterial blood flow and the aneurysm wall. Thus, the type and extent of wall motion may reflect the degree of damage and vulnerability to aneurysmal walls (28). It has been reported that irregular pulsation observed on 4D-CTA may be a dependable indicator for early detection of impending aneurysm rupture and locating the rupture point (11). Nonetheless, few studies have quantified aneurysm dynamic changes over the cardiac cycle. In line with our previous study (29), we found that small UIAs (size < 7 mm) with irregular pulsation exhibited significant degrees of morphological change during the cardiac cycle when compared to larger aneurysms, which tend to show an overall dilation. It is acknowledged that aneurysm rupture occurs when the impact of blood flow on the aneurysm wall exceeds its capacity. The larger the aneurysm, the higher the risk of rupture (30). This interesting paradox highlights a current controversy: the aneurysm size, the most commonly used factor in clinical practice, cannot be relied on alone to evaluate aneurysm rupture risk. Furthermore, irregular pulsation during the cardiac cycle may serve as a promising factor in estimating the rupture risk of small UIAs.

Accurate segmentation and extraction of patient-specific 3D models from medical images is a crucial step in CFD simulation. The output of the geometric model can be influenced by the manipulation of the multimodal images and the subjective bias of the operators, which may impact the hemodynamic results. DSA is considered the gold standard for diagnosing intracranial aneurysms, with high spatial resolution and the ability to provide dynamic blood flow information. Still, the invasive nature of examination limits its widespread clinical use. Cancelliere et al. (31) compared the geometric and hemodynamic features of 16 aneurysm models extracted from DSA and 4D-CTA. They found that the ability of 4D-CTA to characterize the geometry and hemodynamics of aneurysm models was satisfactory (31). Their study results established a foundation for the reliability of the experimental methods employed in the present study. 4D-CTA was used to acquire CT data of aneurysms at 5% intervals throughout the cardiac cycle, and 20 corresponding 3D aneurysm models were reconstructed for CFD simulation. The simulation outcomes are dependable and informative.

### Limitations

This study has several limitations. First, the uncommon use of 4D-CTA for routine clinical examination of IAs resulted in challenges in obtaining data, leading to limited sample size and potential selective bias. Four types of aneurysms based on RRS and cardiac cycle-related irregular pulsation were proposed in this study. Still, the conclusions may not be universally applicable due to the limited number of cases. Secondly, due to the absence of patient-specific TCD data, this study failed to accurately simulate aneurysm hemodynamics at different time phases of the cardiac cycle, which may compromise the investigation of the correlation between irregular pulsation and hemodynamics, consequently restricting the depth of the discussion and the broader applicability of the study findings. Additionally, this is a single-center retrospective study, necessitating follow-up studies to validate the clinical significance of irregular pulsation in assessing rupture risk. Last but not least, the lack of a universally accepted standard for quantifying aneurysm pulsation and the subjective nature of determining irregular pulsation could also impact the study results.

## Conclusion

Regardless of the assessed rupture risk by RRS for UIAs, irregular pulsation during the cardiac cycle may indicate an increased risk of rupture, especially for small aneurysms. The localized area of the aneurysm wall, where irregular pulsation occurs, could be a potential rupture point due to its vulnerable structure. This study would aid in comprehending the dynamic changes of UIAs during the cardiac cycle and the associated hemodynamic mechanisms, to pave the way for the clinical significance of aneurysm dynamics in assessing rupture risk.

## Data availability statement

The original contributions presented in the study are included in the article/supplementary material, further inquiries can be directed to the corresponding author.

## Ethics statement

The studies involving human participants were reviewed and approved by the Institutional Review Board of Changhai Hospital. Written informed consent from the patients/participants or patients/participants’ legal guardian/next of kin was not required to participate in this study in accordance with the national legislation and the institutional requirements.

## Author contributions

SC: Conceptualization, Data curation, Formal analysis, Investigation, Methodology, Software, Visualization, Writing – original draft, Writing – review & editing. WZ: Data curation, Software, Validation, Writing – review & editing. YC: Funding acquisition, Project administration, Supervision, Writing – review & editing. GW: Funding acquisition, Project administration, Supervision, Writing – review & editing. NL: Conceptualization, Data curation, Methodology, Project administration, Resources, Supervision, Validation, Writing – review & editing.
